# A Systematic Review of Nutritional Interventions on Key Cytokine Pathways in Rheumatoid Arthritis and Its Implications for Comorbid Depression: Is a More Comprehensive Approach Required?

**DOI:** 10.7759/cureus.28031

**Published:** 2022-08-15

**Authors:** Michelle Lanspa, Breanne Kothe, Myla R Pereira, Marc M Kesselman, Stephanie N Petrosky

**Affiliations:** 1 College of Medicine, Dr. Kiran C. Patel College of Osteopathic Medicine, Nova Southeastern University, Fort Lauderdale, USA; 2 Rheumatology, Dr. Kiran C. Patel College of Osteopathic Medicine, Nova Southeastern University, Fort Lauderdale, USA; 3 Nutrition, Dr. Kiran C. Patel College of Osteopathic Medicine, Nova Southeastern University, Fort Lauderdale, USA

**Keywords:** interleukin, das-28, crp, depression, comorbid conditions, inflammatory, diet, nutrition, cytokines, rheumatoid arthritis

## Abstract

Rheumatoid arthritis (RA) is associated with both local and systemic inflammatory processes via the aberrant regulation of inflammatory pathways and imbalances in several mediators of inflammation. Cytokines, tumor necrosis factor-alpha (TNF-α), interleukin (IL)-1B, IL-6, IL-17, IL-18, rheumatoid factor, anti-cyclic citrullinated protein, C-reactive protein (CRP), and erythrocyte sedimentation rate (ESR) have been used in diagnosing and tracking the progression of RA. The primary objective of this review is to identify and summarize which specific dietary patterns and nutritional interventions go beyond symptom management to improve the response to known inflammatory cytokines and possibly decrease markers of inflammation in the RA disease process. Analysis of the 41 identified publications demonstrated that certain dietary patterns, the consumption of specific macronutrients, and supplementation with herbals or other compounds have shown some effect on improving cytokine profiles in patients with RA.

This review illustrates the importance of proper patient education on the anti-inflammatory and potential protective impacts substantial dietary change may have on the disease progression and symptoms of RA. Identifying nutritional interventions and dietary patterns that improve the inflammatory cytokine profile, and therefore disease progression and inflammatory comorbidities of RA will help further focus research on treatments that may provide a better overall improvement in quality of life for RA patients by focusing on the root cause inflammatory processes that affect not only joint destruction but also depression-rated disability. This review further notes that while depression is commonly found in patients who suffer from chronic illnesses, it is especially prevalent in the RA population. The pathology of depression is associated with systemic inflammation, which is a known outcome of RA and may explain this strong association. Cytokines IL-6, IL-1, and TNF-α, known mediators involved in the progression of RA, are strongly associated with stress-related disorders including depression and anxiety. The presence of these cytokines is also correlated with the severity and duration of depression. This may signal a potential use of cytokines in diagnosing and following the progression of depression not only in patients with RA but also others. Given the statistics presented on depression and suicide in patients with RA, and the shared inflammatory pathway between the two diseases, depression and suicide screening scales should be included along with analysis of inflammatory markers and disease activity scores (DAS) in any future RA study.

## Introduction and background

The inflammatory profile of rheumatoid arthritis (RA)

RA is a chronic disease characterized by the autoimmune destruction of joints and synovial tissues, which results in pain, deformity, and reduced quality of life in patients with RA [1]. Because of the chronic pain patients with RA endure, the mainstay of treatment aims to reduce inflammation to both suppress symptoms and prevent further joint damage whenever possible [1]. RA is associated with both local and systemic inflammatory processes via the aberrant regulation of inflammatory pathways and imbalances in several mediators of inflammation which are looked at in detail in this study [2]. These systemic inflammatory processes link RA with other inflammatory comorbidities such as cardiovascular disease, malignancy, lung disease, osteoporosis, and neuropsychiatric diseases including depression [3-4]. Due to the expense and side effect profiles of drug regimens for pharmaceutical disease management, [1], and the increasing number of publications addressing the role of nutrition in RA treatment [1,5-6], and the correlation of RA with depression, [3,7-10], the primary objective of this review was to identify and summarize which specific dietary patterns and nutritional interventions go beyond symptom management to improve the response to known inflammatory cytokines and other inflammatory markers in the RA disease process. 

Cytokines are chemical messengers in the body that are secreted by the immune system and can influence communication between cells [11]. Cytokines including tumor necrosis factor-alpha (TNF-α), interleukin (IL)-1B, IL-6, IL-17, and IL-18, as well as inflammatory markers like C-reactive protein (CRP) and erythrocyte sedimentation rate (ESR) that have been used in diagnosing and tracking the progression of RA [11-12]. In many immune reactions and disease processes, TNF-α, IL-6, and IL-1B increase inflammation and leukocyte recruitment, while counter-regulatory cytokines IL-4 and IL-10 decrease inflammation [11]. Dysregulation and imbalance of these systems is the mechanism behind the autoimmune dysfunction seen in RA [11]. Activated macrophages in the joint tissues of RA patients secrete various pro-inflammatory cytokines [11,13]. TNF-α activates fibroblasts and IL-1B causes fibroblast proliferation [13]. This chain of reactions subsequently activates transcription factor nuclear factor-kB (NF-kB), which is one of the main inflammatory pathways seen in RA [13]. In this pathway, the receptor activator of nuclear factor kappa-B ligand (RANKL), which via increased osteoclast activity is involved in bone regeneration and remodeling, is both triggered by and produces high levels of IL-1, TNF-α, and IL-6 [13]. Increased NK-kB expression also results in increased levels of metalloproteinases (MMPs), specifically MMP-2 and MMP-9, which are the enzymes responsible for breaking down cartilage in the synovial fluid of RA patients [11]. Additionally, IL-18 is found in relatively higher concentrations in the synovial tissues of RA patients, which, also via activation of NF-κB, induces interferon-gamma (IFN-𝛾) production from T-cells and natural killer cells in synovial tissues [14]. Finally, studies have shown that IL-17 expression and serum concentrations of IL-6 correlate with RA clinical disease activity [15-16]. IL-17, in addition to provoking the production of pro-inflammatory cytokines IL-6 among others; also encourages Th17 immune cell differentiation, a pro-inflammatory immune pathway, which in patients with RA increases RANKL expression and subsequent osteoclastic activity [17]. In summary, the cytokine processes responsible for disease progression and inflammation in RA involve complex feedback mechanisms principally among interleukins IL-1B, IL-6, IL-17, IL-18, and TNF-α, which is why this review has sought out dietary patterns and nutritional interventions that have attempted to measure impact upon these pathways. 

Impact of diet on the inflammatory cytokines of RA 

The impact of various dietary patterns and nutritional interventions on the disease activity in RA has been investigated by several recent systematic reviews [1,5-6]. The findings from these previous publications support the link between various dietary components, inflammatory markers, and disease activity in RA [1,5-6]. For example, elimination diets, which focus on the removal of common inflammatory foods such as dairy, wheat, and refined sugars, have been shown to lower serum CRP, ESR, IL-1B, and TNF- a, while subsequent food challenges showed increase in these inflammatory markers and subsequent worsening of RA symptoms [1,5]. Other studies have shown that supplementation with high doses of polyunsaturated fatty acids (PUFAs) reduces ESR, CRP, and IL-1B levels while improving Disease Activity Score-28 (DAS-28) scores and quality of life measures in patients with RA [5,6]. Other established findings further supporting the role of dietary intervention in the management of disease in patients with RA include evidence for lowered disease activity with interventions including the Mediterranean diet, flavonoid-containing spices (ginger powder, cinnamon powder, saffron), antioxidants (quercetin and ubiquinone), and probiotics [1,6]. While previous reviews have established the important role diet plays in the management of RA, they have not focused on specific cytokine pathways, nor have they addressed inflammatory comorbid conditions like depression. 

RA and depression

While depression is commonly found in patients who suffer from chronic illnesses, it is especially prevalent in the RA population, up to 42% [8,10]. Compared to healthy matched controls, patients with RA are more than twice as likely to develop depression [3,9]. This has important consequences for patient quality of life, as studies have shown that patients with RA and comorbid depression are more likely to be unemployed (30% versus patients with RA without depression symptoms) and that patients with RA and depression generally display higher DAS and levels of functional disability, with disability correlating to the severity of depression [8,18]. Additionally, Timonen et al. (2003) found reports of suicidal ideations in 14% of female patients with RA and 3% of male patients with RA in a prospective 13-year cohort study [19]. In patients with RA, 52.6% of suicides are by females versus 18.2% in victims who had neither RA nor osteoarthritis (OA), and the median age of completion was 61.7 years old versus 41.3 years old in victims with neither RA nor OA [19]. The pathology of depression is associated with systemic inflammation, which is a known outcome of RA and may explain this strong association [10]. Cytokines IL-6, IL-1, and TNF-α, known mediators involved in the progression of RA, are strongly associated with stress-related disorders including depression and anxiety [7,10]. The presence of these cytokines is also correlated with the severity and duration of depression, marking a possible signal to the potential use of cytokines in diagnosing and following the progression of depression not only in patients with RA but also in others [10]. This is where the present review of literature postulates the potential for nutrition and dietary-based interventions playing a role in improving cytokine profiles in patients with RA not only in the hopes of improving traditional disease progression markers focused solely on the musculoskeletal system but also of improving other cytokine-mediated comorbidities, especially depression.

Objectives

While various dietary patterns and nutritional interventions, as well as supplement studies, have been carried out to propose alternative treatments that might augment traditional pharmaceutical therapy for individuals with RA [5-6,20], given the importance of inflammatory cytokines on the disease progression in RA, on disabling comorbidities like depression, and on their use as biomarkers for disease activity, a new look at nutrition and diet studies was warranted with cytokines and other inflammatory markers specifically in mind. Focusing on a broader cytokine profile than those included in the commonly used DAS-28 for RA (CRP and ESR) allows for a better understanding of how nutritional interventions or dietary patterns directly influence the chronic inflammatory processes of RA. It also allows for more questions to be asked regarding the effect of such interventions on comorbid inflammatory conditions in patients with RA, which share many of the same inflammatory pathways and markers. In summary, identifying nutritional interventions and dietary patterns that improve the inflammatory cytokine profile, and therefore disease progression and inflammatory comorbidities of RA will help further focus research on treatments that may provide a better overall improvement in quality of life for RA patients by focusing on root cause inflammatory processes that affect not only joint description but also depression-rated disability.

## Review

Methods

Search Strategy 

A computer-assisted systematic literature review was performed using PubMed for primary source research articles examining nutritional interventions in the cytokine profiles of patients with RA. The PubMed word search included “rheumatoid arthritis” and “nutrition” or “diet” and “c-reactive protein” or “IL-1” or “IL-6” or “IL-17” or “IL-18” or “cytokines.” Articles were filtered to include those published between 2016 and 2022 to improve efficacy of the review and capture the most recent and prominent research. The reference list of retrieved articles was also considered when found to be relevant and if those additional articles fit the search criteria but were not discovered through the initial search. The relevance of these articles was assessed through a hierarchical approach, with evaluation first of the title, then the abstract, followed by the full manuscript. For any articles not freely available, access was gained through the Nova Southeastern University library. 

Selection Criteria 

**Figure 1:**
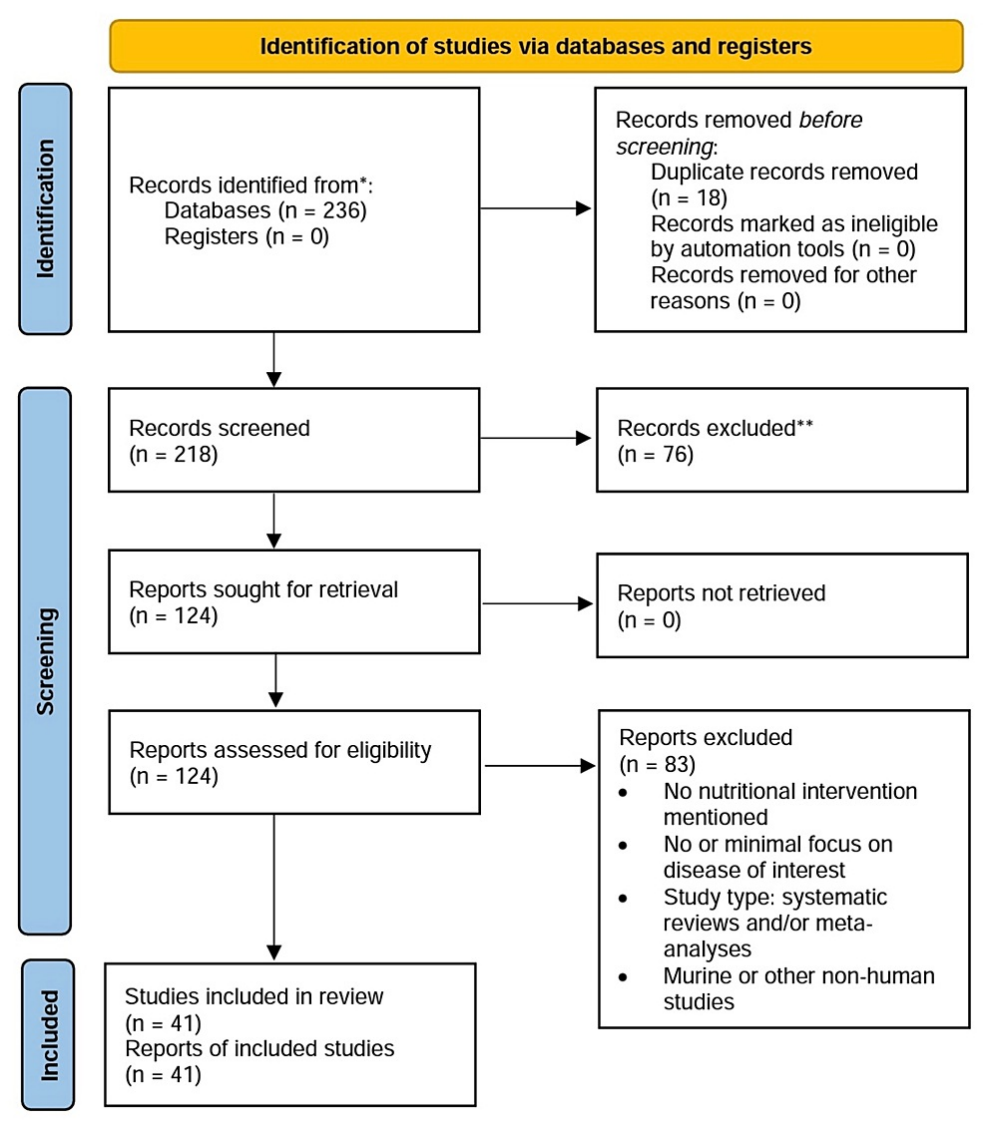
Selection process flow diagram [21]

Studies were considered eligible if they examined specific nutritional interventions in the disease modification of RA and measured the intervention’s effect on one or multiple serum cytokines. Eligible study designs included randomized control trials (RCT), prospective and retrospective cohort studies, cross-sectional studies, case studies, and other studies on human patients and/or human cell lines. Exclusion criteria included meta-analyses, systematic reviews, editorials, non-human studies, and lack of access to the available text. A flow diagram was developed using the Preferred Reporting Items for Systematic Reviews and Meta-Analyses (PRISMA) 2020 outline [21] (Figure 1).

Results

A total of 236 articles were identified from the initial electronic database search. Seventy-six articles were excluded based on lack of relevance found during the title and abstract screening, and 18 duplicates were removed. Of the remaining 124 articles, 83 were excluded for no nutritional intervention mentioned, minimal focus on RA, ineligible study design (based on exclusion criteria), or lack of access to the full text. A total of 41 articles remained for inclusion in this review (Table 1). Most of the publications identified (35 of 41) analyzed dietary patterns or dietary supplements, and the remaining six looked at herbal or other supplements. None of the articles measured any improvements or worsening of symptoms of depression as a result of the studied intervention(s). 

**Table 1:** Summary of nutrition interventions and outcomes CRP = c-reactive protein; DAS = disease activity score; ESR = erythrocytes sedimentation rate; IL = interleukin; MMP = matrix metalloproteinases; PUFAs = polyunsaturated fatty acids; RA = rheumatoid arthritis; RCT = randomized control trial; TNF-α = tumor necrosis factor alpha; HOMA-b = homeostatic model assessment of beta-cell function; HOMA-IR = homeostatic model assessment of insulin resistance.

	Author	Intervention category	Study type	Intervention(s)	n#	Cytokine profile improvements or other results
1	Guagnano et al. (2021) [22]	Diet	RCT	“Private” diet eliminating meat, gluten and lactose for 3 months	n=40	Significant decrease in pain and DAS-28 scores, in addition to an improvement in the overall state of physical and mental health; decreases in diastolic (p = 0.025) and systolic (p = 0.003) arterial pressure and number of circulating leukocytes (p < 0.003), neutrophils (p < 0.006), and the level of CRP also observed
2	Arablou et al. (2019) [23]	Diet	Cross sectional	Dietary intake of vitamins C and E, zinc, magnesium, copper, selenium	n=87	Intakes of vitamin E, zinc, and magnesium in patients were significantly lower and intakes of copper and selenium were significantly higher in patients with RA than recommended daily allowances (p < 0.05); vitamin C intake was associated with decreased IL-1β, zinc intake was associated with decreased IL-2, and magnesium intake was associated with decreased levels of both IL-1β and IL-2 (p< 0.05); vitamin E and copper increased catalase expression, an enzyme largely involved with anti-inflammatory pathways (p< 0.05)
3	Tandorost et al. (2021) [24]	Diet	Case control	Dietary Inflammatory Index (DII)	n=200	Mean DII score was higher in the RA patients as compared with that in the controls (0.66 ± 0.23 vs. -0.58 ± 0.19, p = 0.002); patients with higher DII had significantly higher CRP, TNF-α, DAS-28 scores and number of tender joints
4	McGarrity-Yoder et al. (2021) [25]	Diet	Case control	Dietary quality evaluation	n=50	Age (p = 0.015) and gender (p = 0.003) were associated with higher diet quality; participants with lower diet quality had significantly higher pain (B = -0.396, p = 0.022) and ESR scores (p = 0.019)
5	Vadell et al. (2020) [26]	Diet	RCT	Anti-inflammatory Diet In Rheumatoid Arthritis (ADIRA) Diet	n=44	DAS-28-ESR significantly decreased (p = 0.012) and was significantly lower after the intervention than after the control period in the participants who completed both periods (p = 0.04)
6	Bärebring et al. (2018) [27]	Diet	Cross sectional	Diet quality	n=66	Poor diet quality as defined by the Swedish National Food agency (diet with low intake of fish, shellfish, whole grain, fruit and vegetables and high intake of sausages and sweets) associated with higher CRP (p = 0.044) and ESR (p = 0.002) levels in patients with RA
7	Picchianti Diamanti et al. (2020) [28]	Diet	RCT	Mediterranean diet (MD)	n=60	Patients with high adherence to MD had a significantly lower CRP (p < 0.037) and DAS-28 (p < 0.034) than the 40 patients with low/moderate adherence to MD; a healthier gut microbiota composition was observed in the high adherence group
8	Assar et al. (2020) [29]	Diet	Observational human study	Diurnal fasting for 1 month	n=28	Significant decrease in visual analogue pain scores, tender and swollen joint counts (p=0.02), DAS-28 (p=0.003) and ESR; CRP did decrease however it was not statistically significant
9	Nessib et al. (2020) [30]	Diet	Prospective study	Diurnal fasting for 1 month	n=56	RA patients who participated in diurnal fasting of Ramadan showed significant improvement in DAS-28-CRP (p = 0.001) and DAS-28-ESR (p < 0.001) when compared with patients who did not participate in the fast
10	Naghashian et al. (2019) [31]	Diet	Cross sectional	Anthropometrics, macronutrient consumption	n=77	Weight, BMI, and waist circumference correlated with the activity of RA and the concentrations of CRP and ESR went up in tandem with BMI (r=0.36 and 0.31; p<0.01); asymmetric dimethylarginine but not nesfatin-1 (adipokines), was associated with increased BMI and disease activity in RA patients; higher protein intake correlated to higher CRP and ESR while higher carbohydrate intake correlated to higher CRP and lower nesfatin-1 levels
11	Dürholz et al. (2020) [32]	Diet	RCT	Supplementation with high fiber 30g bars daily for 15 days and 30 days	n=39	Increased anti-inflammatory short-chain fatty acids (p < 0.001), decreased proarthritic cytokine concentrations, and a durable shift in the Firmicutes-to-Bacteroidetes ratio (p < 0.05)
12	Jung et al. (2019) [33]	Diet	Human cell line study	Naive CD4+ T cells cultured in 10, 20, 40, and 60 mM NaCl solutions for 3 days	n=17	NaCl can aggravate arthritis by affecting Th17 differentiation (p<0.05)
13	Scrivo et al. (2017) [34]	Diet	Other human study	Low sodium diet as defined as <5g a day	n=15	Reduction in serum levels of both transforming growth factor beta (TGFβ) (p = 0.0016) and IL-9 (p = 0.0007)
14	De Pablo et al. (2017) [35]	PUFAs	Nested case-control	Erythrocyte levels of n-6 polyunsaturated fatty acid linoleic acid	n=354	Inverse relationship with erythrocyte levels of the n-6 PUFA linoleic acid specifically with risk of RA development (OR 0.29; 95% CI 0.12 to 0.75; p for trend 0.01)
15	Ghaseminasab et al. (2022) [36]	PUFAs	RCT	30g flaxseed supplement per day for 12 weeks	n=120	Decreased DAS-28 scores (- 0.87 ± 1.11 vs. - 0.24 ± 0.78; p = 0.014), pain severity (p ≤ 0.001), morning stiffness (p < 0.05), and disease feeling (p < 0.01) compared to regular diet group but no difference in ESR, CRP, anti-cyclic citrullinated peptide, or rheumatoid factor
16	Lourdudoss et al. (2018) [37]	PUFAs	Prospective case-control	Dietary consumption of PUFAs, tracked for 3 months	n=591	Omega-3 PUFA consumption was inversely associated with (odds ratio [OR] 0.57 [95% confidence interval (95% CI) 0.35-0.95]), and the omega-6:omega-3 ratio was directly associated with (OR 1.70 [95% CI 1.03-2.82]), unacceptable and refractory pain, but not with inflammatory pain or systemic inflammation
17	Tedeschi et al. (2018) [38]	PUFAs	Cross sectional	Dietary recall of average weekly servings of fish	n=176	Significantly lower combined DAS-28-CRP scores (difference -0.49 [95% CI -0.97, -0.02]) when compared to RA patients who ate fish never or <1 time/month, and for each additional serving of fish per week, DAS-28-CRP was significantly reduced by 0.18 (95% CI -0.35, -0.004)
18	Aryaeian et al. (2021) [39]	Flavanoids	RCT	1000 mg black barberry extract for 12 weeks	n=80	No significant effect on IL-2 and IL-4 cytokines, IL-17 levels decreased significantly after the intervention while IL-10 had a significant increase in this group (p > .05)
19	Matsumoto et al. (2021) [40]	Flavanoids	RCT	508.5 mg of brazillian propolis daily for 24 weeks	n=80	No significant differences in DAS-28-ESR, CRP, simplified disease activity index, and clinical disease activity index after 24 weeks of intervention, no significant changes in synovitis, activities of daily living, quality of life, changes in cytokine levels (Group P vs Group C, effect: 0.14, 95% confidence interval: -0.21 to 0.49, p = 0.427)
20	Shishehbor et al. (2018) [41]	Flavanoids	RCT	4 capsules of 500 mg cinnamon powder daily for 8 weeks	n=36	Significant decrease of serum levels of CRP and TNF-α (p < 0.001), significant reduction in DAS-28, visual analogue scale, and tender and swollen joints counts (p < 0.001), no significant decrease in ESR
21	Mateen et al. (2019) [42]	Flavanoids	Human cell line study	Cinnamaldehyde and eugenol on peripheral blood mononuclear cells	N/A	Significant dose-dependent declines in TNF-α and IL-6 (p<0.05), and amelioration of reactive oxygen species formation, bio molecular oxidation, and antioxidant defense response (p<0.05)
22	Fatel et al. (2021) [43]	Flavanoids & PUFAs	Prospective case-control	3 g of fish oil n-3 fatty acids and 500 mL of reduced-calorie cranberry juice daily	n=62	Fish oil only group showed improvements in DAS28-CRP (p=0.0261) and adiponectin (p=0.0239), but when consuming fish oil supplements together with cranberry juice, there were statistically significant reductions in ESR (p=0.033), CRP (p=0.002), DAS-28-CRP (p=0.001), adiponectin (p=0.021), and IL-6 (p=0.045) levels compared to controls
23	Javadi et al. (2019) [44]	Flavanoids	RCT	40 mg of Curcumin nanomicelle 3 times daily for 12 weeks	n=30	No significant decrease in the DAS-28, tender joint count, swollen joint count, and ESR after intervention (p = .885 and p = .162)
24	Pourhabibi-Zarandi et al. (2022) [45]	Flavanoids	RCT	500mg of curcumin daily for 8 weeks	n=48	Significantly decreased insulin resistance, ESR, CRP, triglycerides, weight, body mass index, and waist circumference of RA patients (p < .05 for all)
25	Du et al. (2019) [46]	Flavanoids	Human cell line study	3'3-Diindolylmethane (DIM)	N/A	Inhibited proliferation, migration and invasion of RA fibroblast-like synoviocytes in vitro, significantly decreased TNF-α-induced increases in the mRNA levels of MMP-2, MMP-3, MMP-8, and MMP-9; as well as the proinflammatory factors IL-6, IL-8, and IL-1β
26	Rosillo et al. (2019) [47]	Flavanoids	Human cell line study	Polyphenolic extract from extra virgin olive oil	N/A	Inhibited IL-1β-induced MMPs, TNF-α and IL-6 production (p<0·001); IL-1β-induced cyclo-oxygenase-2 and microsomal prostaglandin synthase-1 up-regulations were down-regulated (p<0·001); IL-1β-induced MAPK phosphorylation and Nuclear Factor kB (NF-κB) activation were also significantly decreased (p<0·001)
27	Moosavian et al. (2020) [48]	Flavanoids	RCT	1,000 mg of garlic daily for 8 weeks	n=70	Significant decreases in CRP (p = .018), TNF- α (p < .001), swollen joint count, pain intensity, tender joint count, DAS-28, and fatigue (p < .001 for all)
28	Ghavipour et al. (2017) [49]	Flavanoids	RCT	2 capsules of 250 mg pomegranate extract daily for 8 weeks	n=55	Significantly decreased DAS-28 (P<0.001), pain intensity (P=0.003), and ESR (P= 0.03); Health Assessment Questionnaire score (p=0.007) and morning stiffness (p=0.04) also signficantly decreased; glutathione peroxidase concentrations (p<0.001) increased; no differences in MMP3 and CRP levels between intervention and control groups
29	Hamidi et al. (2020) [50]	Flavanoids	RCT	100 mg/day of saffron supplement for 12 weeks	n=66	Sigificantly decreased number of tender (-1.38 ± 1.66 vs. 0.10 ± 0.40, p < .001) and swollen (-2.12 ± 2.34 vs. 0.63 ± 2.79, p < .001) joints, pain intensity (-18.36 ± 15.07 vs. -2.33 ± 5.04), p < .001), and DAS-28 (-0.75 ± 0.67 vs. 0.26 ± 0.77, p < .001); Physician Global Assessment and ESR significantly improved (24.06 ± 12.66 vs. 32.00 ± 14.75, p = 0.028); CRP, TNF-α, interferon gamma, and malondialdehyde significantly decreased (12.00 ± 7.40 vs. 8.82 ± 7.930, p = .004)
30	Helli et al. (2019) [51]	Flavanoids	RCT	200-mg/day sesamin supplement for 6 weeks	n=44	Serum levels of hyaluronidase and MMP-3 decreased significantly (p=0.045 and 0.039, respectively); serum levels of CRP, TNF-α, and cyclooxygenase-2 in intervention group significantly decreased (p=0.046, 0.039, and 0.004, respectively); significant reduction in the number of tender joints and severity of pain
31	Cannarella et al. (2021) [52]	Probiotics	RCT	L. acidophilus, L. casei, L. lactis, B. lactis, and B. bifidum for 60 days	n=42	Improvements in inflammatory profiles with reductions in white blood cell count (p=0.012), TNF-α (p=0.004), and IL-6 (Ip=0.038) levels, but no difference in IL-10 levels, adiponectin, CRP, ESR, ferritin, or DAS-28
32	Hong et al. (2021) [53]	Probiotics	Human cell line, in vitro study	Recombinant B. bifidum	In vitro	Significantly higher IL-10 levels than those from food grade bacteria and inhibited levels of IL-6, IL-8, and TNF-α
33	Jeong et al. (2021) [54]	Probiotics	Human cell line, in vitro study	B. longum	In vitro	Significant inhibition of Th17 cells and IL-17 related genes, as well as several other proinflammatory mediators
34	Zamani et al. (2016) [55]	Probiotics	RCT	L. acidophilus, L. casei and B. bifidum dophilus for 8 weeks	n=60	Decreased serum CRP (-6.66 ± 2.56 vs. +3.07 ± 5.53 mg/L, p < 0.001), insulin levels (-2.0 ± 4.3 vs. +0.5 ± 4.9 μIU/mL, p = 0.03), and improved DAS-28 (-0.3 ± 0.4 vs. -0.1 ± 0.4, p = 0.01) and HOMA-b score (-7.5 ± 18.0 vs. +4.3 ± 25.0, p = 0.03) scores but no improvement in HOMA-IR score, lipid profiles, or other bio makers of oxidative stress levels
35	Zamani et al. (2017) [56]	Probiotics	RCT	Symbiotic supplement for 8 weeks	n=54	Reductions in CRP (-1427·8 (sd 3267·2) v. +2833·4 (sd 5639·7) ng/ml, P=0·001), HOMA-IR (-0·5 (sd 1·0) v.+0·1 (sd 1·1), P=0·03), HOMA-B scores (-9·4 (sd 17·9) v. +3·3 (sd 18·9), P=0·01), and improvements in DAS-28 scores (-1·6 (sd 0·8) v. -0·3 (sd 0·5), P<0·001) and plasma nitric oxide levels (+0·8 (sd 4·4) v. -2·6 (sd 4·5) µmol/l, P=0·008)
36	Kheirouri et al. (2016) [57]	Herbals	RCT	Nigella sativa extract, 500mg twice daily for 2 months	n=43	Statistically significant decrease in CRP serum levels (p = 0.007) and DAS-28 scores (p = 0.02) compared to controls
37	Mirtaheri et al. (2022) [58]	Herbals	RCT	Stachys schtschegleevii tea, 2.4g daily for 8 weeks	n=44	Marked reductions in DAS-28 scores (SSC: -32.44% vs. placebo: -22.32%, mean differences= -0.41, p<0.05)) and serum MMP-3 levels (SSC: -20.59% vs. placebo: 1.29%, p<0.05)
38	Sun et al. (2016) [59]	Herbals	RCT	Xinfeng supplement, 3 pills three times a day for 2 months	n=80	Decreased levels of ESR, CRP, and DAS-28 decreased (p<0. 05)
39	Hashemi at al. (2019) [60]	Others	RCT	N-Acetyl Cysteine, 600mg twice daily for 12 weeks	n=42	Reduced levels of malondialdehyde, nitric oxide, and total thiol groups compared to control
40	Prescha et al. (2019) [61]	Others	Retrospective case-control	Serum silicon levels	n=244	Mixed results on redox and inflammatory statuses, further studies needed
41	Shishavan et al. (2019) [62]	Others	RCT	Vitamin K1, 10mg daily for 8 weeks	n=58	No significant reduction in DAS-28 scores or serum IL-6 levels after adjusting for relevant confounders

Discussion

From the analyzed publications, four main overarching themes stand out: 1. Poor diet quality and/or poor adherence to anti-inflammatory diets in patients with RA results in increased inflammatory markers and pain scores [22-31]; 2. Dietary consumption of antioxidants, PUFAs, and fiber have preferable disease outcomes in patients with RA, whereas salt does not [32-38]; 3. Flavanoid supplementation, particularly with barbary extract, cinnamon, cranberry juice, curcumin, garlic, saffron, pomegranate extract, sesamin, and diindolylmethane (DIM) in patients with RA has shown promising results in reducing inflammatory cytokines active in the RA disease process [39-51]; and 4. Probiotic supplementation may be beneficial in ameliorating the profile of gut microbiota in patients with RA, also resulting in decreased disease activity [52-56].

Dietary Patterns 

A significant number of the publications (26 of 41) identified focused on dietary evaluations or interventions. The studies looked at various factors like anthropometrics and diet quality, in addition to identifying various interventions analyzing the effect of fasting diets, other specific diets, and intake of salt, fiber, antioxidants, and PUFAs on the inflammatory status of patients with RA. Generally, poor diet quality and diets considered inflammatory has been shown to worsen cytokine profiles and DAS [22,24-28]. Several studies in this review [24-28], found that diets defined as “poor” in quality (diets with low intake of fish, shellfish, whole grains, fruits, and vegetables and high intake of smoked meats and sweets), diets with higher dietary inflammatory index (DII) scores, diets with low adherence to the Anti-inflammatory Diet in Rheumatoid Arthritis (ADIRA) protocol, or diets with low adherence to the Mediterranean diet are associated with poorer outcomes including higher CRP and ESR levels and increased pain levels. Elimination diets excluding foods known to trigger inflammatory processes, for example, red meat, gluten, and dairy have also been explored as adjuvant treatment for RA patients, with one study showing a significant reduction in CRP levels, in addition to improved pain scores, lower levels of circulating leukocytes and neutrophils, and improved quality of life in patients with RA compared to controls [22]. 

In addition to dietary content, several other studies identified additional considerations in dietary patterns like timing and underlying anthropometrics. Intermittent fasting in the setting of diurnal fasting during Ramadan was explored as a potential method for remediating inflammation in RA in two studies [29-30]; both found improvements in inflammatory markers and pain scores during the month of fasting. One study that investigated the relationship between anthropometric measurements concluded that weight, BMI, and waist circumference were positively correlated with disease activity in RA [31]. Serum concentrations of CRP and ESR increased with increasing BMI, suggesting dietary interventions aimed solely at weight loss may positively impact disease activity and overall patient quality of life [31]. In regard to the aforementioned adipokines, ADMA, but not nesfatin-1, was associated with higher BMI and disease activity in RA patients [31]. 

Dietary Consumption of Salt, Antioxidants, and PUFAs

Specific dietary consumption studies included publications evaluating the effect of salt, antioxidants, fiber, and PUFAs consumption [23,32-38]. Recently, the relationship between sodium chloride (NaCl) intake and inflammation has been investigated as a potential modifier of autoimmune diseases such as RA and systemic lupus erythematosus (SLE) [33]. Studies in this review have found that synovial fluid NaCl concentration was associated with enhanced Th17 cell proliferation and resultant increases in disease activity [33] and that patients on a low sodium diet resulted in reduced Th17 cell concentrations and reductions in IL-9 and tumor growth factor beta (TGF-β) after just three weeks [34].

Antioxidants play an important role in the balance between pro- and anti-inflammatory processes within the human body and are the subject of one publication in this review. Dietary recalls revealed that patients with RA generally had higher intakes of selenium and copper, and lower intakes of zinc, vitamin E, and magnesium than recommended guidelines [23]. Adequate vitamin C intake was associated with decreased IL-1β, zinc intake was associated with decreased IL-2, and magnesium intake was associated with decreased levels of both IL-1β and IL-2, whereas deficient intake of vitamin E and copper were noted to decrease catalase expression, an enzyme largely involved with anti-inflammatory pathways [23].

Five of the articles in this sample discuss the role of PUFAs in reducing inflammation in patients with RA. PUFAs, primarily omega-3 long-chain fatty acids eicosapentaenoic acid (EPA) and docosahexaenoic acid (DHA), are precursor molecules in the biosynthesis of anti-inflammatory prostaglandins and leukotrienes and act as inhibitors in the conversion of arachidonic acid to inflammatory prostaglandins and leukotrienes [63]. Omega-6 fatty acid alpha-linolenic acid (ALA) also exhibits similar anti-inflammatory activity [63]. Omega-3 supplementation has also been shown to reduce the synthesis of IL-1 and TNF-α in monocytes and IL-6 in endothelial cells [64]. Additionally, higher omega-6:omega-3 ratios have been correlated with overproduction of proinflammatory cytokines [64]. Three of the five studies evaluated PUFA consumption via dietary recall [33,37-38]. The first study found that subjects who consumed fish, a food high in PUFA content, two or more times a week to have significantly lower combined DAS-28-CRP scores when compared to RA patients who ate fish never or <1 time/month, and that for each additional serving of fish per week, DAS-28-CRP was significantly reduced [38]. The second study found that diets low in omega-3 PUFAs and diets with greater omega-6:omega-3 ratios were directly associated with unacceptable and refractory pain, but not with inflammatory pain or systemic inflammation, suggesting that omega-3 PUFAs might help with pain control in RA [37]. The last PUFA dietary recall study demonstrated an inverse relationship with erythrocyte levels of the n-6 PUFA linoleic acid, specifically with risk of RA development [35].

Supplementation with PUFAs, Fiber, Flavanoids, and Probiotics

A study [43] concerning supplementation with PUFAs showed statistically significant reductions in DAS-28 scores and adiponectin levels compared to controls in the fish oil only group, and interestingly, better results when consuming fish oil supplements together with cranberry juice, resulting in further reductions in ESR, CRP, and IL-6 levels versus fish oil alone. Another PUFA supplement study with flaxseed showed improved DAS-28 scores but no changes in inflammatory markers [36].

Another dietary component under investigation for RA symptom relief is dietary fiber. Supplementation with high fiber bars developed to contain 30 grams of a combination of non-digestible soluble and insoluble carbohydrates of three or more monomeric units given daily over a 30-day period showed increased systemic short-chain fatty acid (SCFA) levels in RA patients, which are known to decrease inflammation by upregulating Treg cells, decreasing T cell proliferation, and decreasing IL-18 concentrations [32].

Flavonoids are a type of polyphenolic secondary metabolites found in plants [33]. They are widely known to exhibit powerful antioxidant, anti-inflammatory, and immunomodulatory properties [65]. Thirteen of the 41 articles analyzed involved supplement and food interventions known to be high in flavanoid concentrations. RCTs evaluating barbary extract, cinnamon, cranberry juice, garlic, saffron, pomegranate extract, sesamin, polyphenolic extract from olive oil, and DIM extract from cruciferous vegetables showed reductions in several inflammatory cytokines (see Table 1 for cytokines measured with p values) [39-51]. There were mixed results with curcumin with one RCT finding no significant improvements, while another did see decreased CRP, ERP, and other inflammatory markers in patients with RA [44-45]. Finally, no effect was seen in the RCT evaluating Brazilian propolis [40].

Five of the 41 articles analyzed involved interventions with probiotics. Patients with RA were observed to have different gut microbiomes from healthy controls [54-55]. These differences in intestinal bacteria have been shown to contribute to impaired insulin metabolism, further perpetuating inflammation and oxidative stress in patients with RA [55]. Additionally, the gut microbiota is closely linked to systemic inflammatory processes due to its manipulation of macrophage activity [66]. Regarding RA pathogenesis specifically, it is hypothesized that luminal bacterial translocation secondary to increased epithelial permeability and exposure to variable categories and amounts of bacterial antigens are potential links between gut dysbiosis and joint inflammation [28]. Two RCTs found that daily treatment with probiotics in patients with RA resulted in decreased serum hs-CRP levels and improved DAS-28 scores [55-56]. Another RCT evaluating a mixed probiotic showed improvements in inflammatory profiles with reductions in white blood cell count, TNF-α, and IL-6 plasma levels, but no difference with placebo in IL-10 levels, adiponectin, CRP, ESR, ferritin, or DAS-28 [52]. Two in vitro interventions with probiotic supplementation showed inhibited levels of IL-6, IL-8, and TNF-α [53] and inhibited Th17 cells and IL-17 related genes as well as several other proinflammatory mediators [54].

Of note, the Mediterranean diet, known for its balanced nutrient profile and various antioxidant and anti-inflammatory properties, has been commonly investigated regarding its impact on patients with RA [28]. In addition to demonstrating improvements in inflammatory markers and pain scores, one study in this review sought to evaluate the effect of the Mediterranean diet on the gut microbiota [28]. Their study reported significant decreases in Lactobacillaceae and Prevotella copri species in patients who were highly adherent to the Mediterranean diet, which indicated a healthier gut microbiota composition compared to the study’s moderate/low adherence counterparts [28]. 

Other Interventions 

Three of the 41 articles identified in this review were RCTs that evaluated the anti-inflammatory benefits of herbal supplements in patients with RA [57-59]. Supplementation with Nigella sativa extract, commonly used in Middle and far Eastern countries as botanical medicine, consumption of stachys schtschegleevii (SSC), a common herbal medicine consumed in the form of tea, and supplementation with Chinese herbal medicine Xinfeng all showed improvements inflammatory markers and DAS [57-59].

The final three studies evaluated do not fit into any of the prior intervention categories. They include human studies on Vitamin K1, n-acetyl cysteine (NAC), and silicon intake [60-62]. Vitamin K1 supplementation, compared to placebo, demonstrated no significant reduction in DAS-28 scores or serum IL-6 levels after adjusting for relevant confounders [62]. Supplementation with NAC, an important facilitator of phase 1 and phase 2 detoxification in the liver that has been shown to improve inflammation profiles, resulted in significantly lower levels of malondialdehyde, NO, and total thiol groups, but no statistically significant difference in serum IL-6, TNF-α, ESR or CRP levels between the control and intervention groups [60]. Finally, silicon, which is a common food additive and also ingested from dietary sources like cereals and certain vegetables, is retained by connective tissues and has been correlated with higher collagen concentrations in the skin and cartilage, improved bone density, and some anti-inflammatory responses; however, hypercalcemia causes oxidative stress, and non-dietary sources of silicon (implants) have been found to increase the risk for autoimmune diseases in humans [61]. The study on silicon found that patients with RA, compared to healthy controls, had significantly higher serum concentrations of silicon, and exhibited a negative correlation between silicon levels and the number of swollen joints and serum IL-6 levels, however, these results were only found in females [66]. The study is overall inconclusive, noting that albumin levels, smoking status, and gender interfered with the results [61].

Final Considerations

None of the articles identified in this review measured any improvements or worsening of symptoms of depression as a result of the studied intervention(s). We propose that further studies on RA interventions should track outcomes in inflammatory and cytokine markers, and utilize validated scales and/or accepted measures of disease activity to evaluate any changes in comorbid inflammatory conditions, especially depression, to best provide comprehensive quality of life improvements for patients with RA.

## Conclusions

Analysis of the 41 identified publications demonstrates that certain dietary patterns, the consumption of specific macronutrients, and supplementation with herbals or other compounds have shown some effect on improving cytokine profiles in patients with RA. This review illustrates the importance of proper patient education on the anti-inflammatory and potential protective impacts substantial dietary change may have on the disease progression and symptoms of RA. More homogenous studies and further analysis measuring the quality of the resulting data would provide more robust suggestions on nutritional and dietary interventions for patients with RA. Given the statistics presented on depression and suicide in patients with RA, and the shared inflammatory pathway between the two diseases, depression and suicide screening scales should be included along with analysis of inflammatory markers and DAS in any future RA study. 

## References

[1] Marquez AM, Evans C, Boltson K, Kesselman M (2020). Nutritional interventions and supplementation for rheumatoid arthritis patients: a systematic review for clinical application, part 1: dieting. Curr Rheumatol Res.

[2] Smolen JS, Aletaha D (2009). Developments in the clinical understanding of rheumatoid arthritis. Arthritis Res Ther.

[3] Jeong H, Baek SY, Kim SW (2017). Comorbidities of rheumatoid arthritis: results from the Korean National Health and Nutrition Examination Survey. PLoS One.

[4] Kłodziński Ł, Wisłowska M (2018). Comorbidities in rheumatic arthritis. Reumatologia.

[5] Philippou E, Petersson SD, Rodomar C, Nikiphorou E (2021). Rheumatoid arthritis and dietary interventions: systematic review of clinical trials. Nutr Rev.

[6] Nelson J, Sjöblom H, Gjertsson I, Ulven SM, Lindqvist HM, Bärebring L (2020). Do interventions with diet or dietary supplements reduce the disease activity score in rheumatoid arthritis? A systematic review of randomized controlled trials. Nutrients.

[7] Chimenti MS, Fonti GL, Conigliaro P (2021). The burden of depressive disorders in musculoskeletal diseases: is there an association between mood and inflammation?. Ann Gen Psychiatry.

[8] Maldonado G, Ríos C, Paredes C (2017). Depression in rheumatoid arthritis. Rev Colomb Reumatol.

[9] Wang SL, Chang CH, Hu LY, Tsai SJ, Yang AC, You ZH (2014). Risk of developing depressive disorders following rheumatoid arthritis: a nationwide population-based study. PLoS One.

[10] Zhang C (2021). Flare-up of cytokines in rheumatoid arthritis and their role in triggering depression: shared common function and their possible applications in treatment (review). Biomed Rep.

[11] Gandhi GR, Jothi G, Mohana T (2021). Anti-inflammatory natural products as potential therapeutic agents of rheumatoid arthritis: a systematic review. Phytomedicine.

[12] Liu E, Perl A (2019). Pathogenesis and treatment of autoimmune rheumatic diseases. Curr Opin Rheumatol.

[13] Ilchovska DD, Barrow DM (2021). An Overview of the NF-kB mechanism of pathophysiology in rheumatoid arthritis, investigation of the NF-kB ligand RANKL and related nutritional interventions. Autoimmun Rev.

[14] Rex DA, Agarwal N, Prasad TS, Kandasamy RK, Subbannayya Y, Pinto SM (2020). A comprehensive pathway map of IL-18-mediated signalling. J Cell Commun Signal.

[15] Dissanayake K, Jayasinghe C, Wanigasekara P, Sominanda A (2021). Potential applicability of cytokines as biomarkers of disease activity in rheumatoid arthritis: enzyme-linked immunosorbent spot assay-based evaluation of TNF-α, IL-1β, IL-10 and IL-17A. PLoS One.

[16] Boyapati A, Schwartzman S, Msihid J (2020). Association of high serum interleukin-6 levels with severe progression of rheumatoid arthritis and increased treatment response differentiating sarilumab from adalimumab or methotrexate in a post hoc analysis. Arthritis Rheumatol.

[17] Omidian Z, Ahmed R, Giwa A, Donner T, Hamad AR (2019). IL-17 and limits of success. Cell Immunol.

[18] Jetha A, Theis KA, Boring MA, Murphy LB, Guglielmo D (2021). Depressive symptoms and the arthritis-employment interface: a population-level study. Arthritis Care Res.

[19] Timonen M, Viilo K, Hakko H (2003). Suicides in persons suffering from rheumatoid arthritis. Rheumatology (Oxford).

[20] Marquez AM, Evans C, Boltson K, Kesselman M (2020). Nutritional interventions and supplementation for rheumatoid arthritis patients: a systematic review for clinical application, part 2: supplementation. Curr Rheumatol Res.

[21] Page MJ, Moher D, Bossuyt PM (2021). PRISMA 2020 explanation and elaboration: updated guidance and exemplars for reporting systematic reviews. BMJ.

[22] Guagnano MT, D'Angelo C, Caniglia D (2021). Improvement of inflammation and pain after three months' exclusion diet in rheumatoid arthritis patients. Nutrients.

[23] Arablou T, Aryaeian N, Djalali M, Shahram F, Rasouli L (2019). Association between dietary intake of some antioxidant micronutrients with some inflammatory and antioxidant markers in active rheumatoid arthritis patients. Int J Vitam Nutr Res.

[24] Tandorost A, Kheirouri S, Moludi J, Seyedmardani S (2021). Association of Dietary Inflammatory Index (DII) with disease activity and inflammatory cytokines in the patients with rheumatoid arthritis. Int J Clin Pract.

[25] McGarrity-Yoder ME, Insel KC, Crane TE, Pace TW (2021). Diet quality and disease activity in rheumatoid arthritis. Nutr Health.

[26] Vadell AK, Bärebring L, Hulander E, Gjertsson I, Lindqvist HM, Winkvist A (2020). Anti-inflammatory Diet In Rheumatoid Arthritis (ADIRA)-a randomized, controlled crossover trial indicating effects on disease activity. Am J Clin Nutr.

[27] Bärebring L, Winkvist A, Gjertsson I, Lindqvist HM (2018). Poor dietary quality is associated with increased inflammation in Swedish patients with rheumatoid arthritis. Nutrients.

[28] Picchianti Diamanti A, Panebianco C, Salerno G (2020). Impact of Mediterranean diet on disease activity and gut microbiota composition of rheumatoid arthritis patients. Microorganisms.

[29] Assar S, Almasi P, Almasi A (2020). Effect of Islamic fasting on the severity of rheumatoid arthritis. JNFH.

[30] Ben Nessib D, Maatallah K, Ferjani H, Kaffel D, Hamdi W (2020). Impact of Ramadan diurnal intermittent fasting on rheumatic diseases. Clin Rheumatol.

[31] Naghashian F, Hosseinzadeh-Attar MJ, Akhlaghi M, Yekaninejad MS, Aryaeian N, Derakhshanian H (2019). The relationship between anthropometric status and rheumatoid arthritis. Exploring the role of nesfatin and asymmetric dimethylarginine. Acta Reumatol Port.

[32] Dürholz K, Hofmann J, Iljazovic A (2020). Dietary short-term fiber interventions in arthritis patients increase systemic SCFA levels and regulate inflammation. Nutrients.

[33] Jung SM, Kim Y, Kim J (2019). Sodium chloride aggravates arthritis via Th17 polarization. Yonsei Med J.

[34] Scrivo R, Massaro L, Barbati C (2017). The role of dietary sodium intake on the modulation of T helper 17 cells and regulatory T cells in patients with rheumatoid arthritis and systemic lupus erythematosus. PLoS One.

[35] de Pablo P, Romaguera D, Fisk HL (2018). High erythrocyte levels of the n-6 polyunsaturated fatty acid linoleic acid are associated with lower risk of subsequent rheumatoid arthritis in a southern European nested case-control study. Ann Rheum Dis.

[36] Ghaseminasab-Parizi M, Nazarinia MA, Akhlaghi M (2022). The effect of flaxseed with or without anti-inflammatory diet in patients with rheumatoid arthritis, a randomized controlled trial. Eur J Nutr.

[37] Lourdudoss C, Di Giuseppe D, Wolk A (2018). Dietary intake of polyunsaturated fatty acids and pain in spite of inflammatory control among methotrexate-treated early rheumatoid arthritis patients. Arthritis Care Res.

[38] Tedeschi SK, Bathon JM, Giles JT, Lin TC, Yoshida K, Solomon DH (2018). Relationship between fish consumption and disease activity in rheumatoid arthritis. Arthritis Care Res.

[39] Aryaeian N, Hadidi M, Mahmoudi M, Asgari M, Hezaveh ZS, Sadehi SK (2021). The effect of black barberry hydroalcoholic extract on immune mediators in patients with active rheumatoid arthritis: a randomized, double-blind, controlled clinical trial. Phytother Res.

[40] Matsumoto Y, Takahashi K, Sugioka Y (2021). Double-blinded randomized controlled trial to reveal the effects of Brazilian propolis intake on rheumatoid arthritis disease activity index; BeeDAI. PLoS One.

[41] Shishehbor F, Rezaeyan Safar M, Rajaei E, Haghighizadeh MH (2018). Cinnamon consumption improves clinical symptoms and inflammatory markers in women with rheumatoid arthritis. J Am Coll Nutr.

[42] Mateen S, Rehman MT, Shahzad S (2019). Anti-oxidant and anti-inflammatory effects of cinnamaldehyde and eugenol on mononuclear cells of rheumatoid arthritis patients. Eur J Pharmacol.

[43] Fatel EC, Rosa FT, Alfieri DF (2021). Beneficial effects of fish oil and cranberry juice on disease activity and inflammatory biomarkers in people with rheumatoid arthritis. Nutrition.

[44] Javadi M, Khadem Haghighian H, Goodarzy S, Abbasi M, Nassiri-Asl M (2019). Effect of curcumin nanomicelle on the clinical symptoms of patients with rheumatoid arthritis: a randomized, double-blind, controlled trial. Int J Rheum Dis.

[45] Pourhabibi-Zarandi F, Rafraf M, Zayeni H, Asghari-Jafarabadi M, Ebrahimi AA (2022). Effects of curcumin supplementation on metabolic parameters, inflammatory factors and obesity values in women with rheumatoid arthritis: a randomized, double-blind, placebo-controlled clinical trial. Phytother Res.

[46] Du H, Zhang X, Zeng Y (2019). novel phytochemical, DIM, inhibits proliferation, migration, invasion and TNF-α induced inflammatory cytokine production of synovial fibroblasts from rheumatoid arthritis patients by targeting MAPK and AKT/mTOR signal pathway. Front Immunol.

[47] Rosillo MÁ, Alarcón-de-la-Lastra C, Castejón ML, Montoya T, Cejudo-Guillén M, Sánchez-Hidalgo M (2019). Polyphenolic extract from extra virgin olive oil inhibits the inflammatory response in IL-1β-activated synovial fibroblasts. Br J Nutr.

[48] Moosavian SP, Paknahad Z, Habibagahi Z, Maracy M (2020). The effects of garlic (Allium sativum) supplementation on inflammatory biomarkers, fatigue, and clinical symptoms in patients with active rheumatoid arthritis: a randomized, double-blind, placebo-controlled trial. Phytother Res.

[49] Ghavipour M, Sotoudeh G, Tavakoli E, Mowla K, Hasanzadeh J, Mazloom Z (2017). Pomegranate extract alleviates disease activity and some blood biomarkers of inflammation and oxidative stress in rheumatoid arthritis patients. Eur J Clin Nutr.

[50] Hamidi Z, Aryaeian N, Abolghasemi J, Shirani F, Hadidi M, Fallah S, Moradi N (2020). The effect of saffron supplement on clinical outcomes and metabolic profiles in patients with active rheumatoid arthritis: a randomized, double-blind, placebo-controlled clinical trial. Phytother Res.

[51] Helli B, Shahi MM, Mowla K, Jalali MT, Haghighian HK (2019). A randomized, triple-blind, placebo-controlled clinical trial, evaluating the sesamin supplement effects on proteolytic enzymes, inflammatory markers, and clinical indices in women with rheumatoid arthritis. Phytother Res.

[52] Cannarella LA, Mari NL, Alcântara CC (2021). Mixture of probiotics reduces inflammatory biomarkers and improves the oxidative/nitrosative profile in people with rheumatoid arthritis. Nutrition.

[53] Hong N, Ku S, Yuk K, Johnston TV, Ji GE, Park MS (2021). Production of biologically active human interleukin-10 by Bifidobacterium bifidum BGN4. Microb Cell Fact.

[54] Jeong Y, Jhun J, Lee SY (2021). Therapeutic potential of a novel bifidobacterium identified through microbiome profiling of RA patients with different RF levels. Front Immunol.

[55] Zamani B, Golkar HR, Farshbaf S (2016). Clinical and metabolic response to probiotic supplementation in patients with rheumatoid arthritis: a randomized, double-blind, placebo-controlled trial. Int J Rheum Dis.

[56] Zamani B, Farshbaf S, Golkar HR, Bahmani F, Asemi Z (2017). Synbiotic supplementation and the effects on clinical and metabolic responses in patients with rheumatoid arthritis: a randomised, double-blind, placebo-controlled trial. Br J Nutr.

[57] Kheirouri S, Hadi V, Alizadeh M (2016). Immunomodulatory effect of Nigella sativa oil on T lymphocytes in patients with rheumatoid arthritis. Immunol Invest.

[58] Mirtaheri E, Khabbazi A, Nazemiyeh H, Ebrahimi AA, Hajalilou M, Shakibay Novin Z, Pirouzpanah S (2022). Stachys schtschegleevii tea, matrix metalloproteinase, and disease severity in female rheumatoid arthritis patients: a randomized controlled clinical trial. Clin Rheumatol.

[59] Sun Y, Liu J, Wan L (2016). Effect of xinfeng capsule on improving pulmonary function in rheumatoid arthritis patients [Article in Chinese]. Zhongguo Zhongxiyi jiehe zazhi.

[60] Hashemi G, Mirjalili M, Basiri Z (2019). A pilot study to evaluate the effects of oral n-acetyl cysteine on inflammatory and oxidative stress biomarkers in rheumatoid arthritis. Curr Rheumatol Rev.

[61] Prescha A, Zabłocka-Słowińska K, Płaczkowska S, Gorczyca D, Łuczak A, Grajeta H (2019). Silicon intake and plasma level and their relationships with systemic redox and inflammatory markers in rheumatoid arthritis patients. Adv Clin Exp Med.

[62] Shishavan NG, Gargari BP, Jafarabadi MA, Kolahi S, Haggifar S, Noroozi S (2018). Vitamin K1 supplementation did not alter inflammatory markers and clinical status in patients with rheumatoid arthritis. Int J Vitam Nutr Res.

[63] Veselinovic M, Vasiljevic D, Vucic V (2017). Clinical benefits of n-3 PUFA and ɤ-linolenic acid in patients with rheumatoid arthritis. Nutrients.

[64] Simopoulos AP (2002). Omega-3 fatty acids in inflammation and autoimmune diseases. J Am Coll Nutr.

[65] Hughes SD, Ketheesan N, Haleagrahara N (2017). The therapeutic potential of plant flavonoids on rheumatoid arthritis. Crit Rev Food Sci Nutr.

[66] Wang J, Chen WD, Wang YD (2020). The relationship between gut microbiota and inflammatory diseases: The role of macrophages. Front Microbiol.

